# Direct and Indirect Suppression of Interleukin-6 Gene Expression in Murine Macrophages by Nuclear Orphan Receptor REV-ERB*α*

**DOI:** 10.1155/2014/685854

**Published:** 2014-10-14

**Authors:** Shogo Sato, Takuya Sakurai, Junetsu Ogasawara, Ken Shirato, Yoshinaga Ishibashi, Shuji Oh-ishi, Kazuhiko Imaizumi, Shukoh Haga, Yoshiaki Hitomi, Tetsuya Izawa, Yoshinobu Ohira, Hideki Ohno, Takako Kizaki

**Affiliations:** ^1^Department of Molecular Predictive Medicine and Sport Science, Kyorin University, School of Medicine, 6-20-2 Shinkawa, Mitaka, Tokyo 181-8611, Japan; ^2^Department of Respiratory Medicine, National Hospital Organization, Ibarakihigashi National Hospital, 825 Terunuma, Tokai-mura, Naka-gun, Ibaraki 319-1113, Japan; ^3^Faculty of Human Sciences, Waseda University, 2-579-15 Mikajima, Tokorozawa, Saitama 359-1192, Japan; ^4^Graduate School of Comprehensive Human Sciences, University of Tsukuba, 1-1-1 Tennodai, Tsukuba, Ibaraki 305-8573, Japan; ^5^Graduate School of Medical Science, Kanazawa University, Kakumamachi, Kanazawa 920-1192, Japan; ^6^Faculty of Health and Sport Science, Doshisha University, Kyotanabe, Kyoto 610-0394, Japan

## Abstract

It is now evident that many nuclear hormone receptors can modulate target gene expression. REV-ERB*α*, one of the nuclear hormone receptors with the capacity to alter clock function, is critically involved in lipid metabolism, adipogenesis, and the inflammatory response. Recent studies suggest that REV-ERB*α* plays a key role in the mediation between clockwork and inflammation. The purpose of the current study was to investigate the role of REV-ERB*α* in the regulation of *interleukin-6* (*il6*) gene expression in murine macrophages. REV-ERB*α* agonists, or overexpression of* rev-erb* *α* in the murine macrophage cell line RAW264 cells, suppressed the induction of *il6* mRNA following a lipopolysaccharide (LPS) endotoxin challenge. Also, *rev-erb* *α* overexpression decreased LPS-stimulated nuclear factor *κ*B (NF*κ*B) activation in RAW264 cells. We showed that REV-ERB*α* represses *il6* expression not only indirectly through an NF*κ*B binding motif but also directly through a REV-ERB*α* binding motif in the murine *il6* promoter region. Furthermore, peritoneal macrophages from mice lacking *rev-erb* *α* increased *il6* mRNA expression. These data suggest that REV-ERB*α* regulates the inflammatory response of macrophages through the suppression of *il6* expression. REV-ERB*α* may therefore be identified as a potent anti-inflammatory receptor and be a therapeutic target receptor of inflammatory diseases.

## 1. Introduction

The human genome contains 48 nuclear hormone receptor genes, comprising a large family of ligand-dependent transcription factors. In contrast with most classic receptors, nuclear hormone receptors modulate transcription by binding directly to DNA, and ligand interactions occur primarily within the cell cytosol or nucleus. Nuclear hormone receptors are now recognized as key intermediaries between the molecular clock machinery and a wide array of physiological processes [1]. In particular, REV-ERB*α*, one of the nuclear hormone receptors encoded by* nr1d1*, is a crucial regulator of lipid, lipoprotein metabolism, and inflammation [1].

REV-ERB*α*, one of the key clock genes, is a part of the clock machinery and plays an important role in maintaining the proper rhythm of circadian timing [2]. REV-ERB*α* binds as a monomer to the retinoic acid receptor-related orphan receptor (ROR) response elements (ROREs) composed of a 6 bp A/T-rich sequence immediately preceding a site with the core motif of (A/G)GGTCA [3]. It also binds as a homodimer to the RevDR2 response element, which is composed of a 6 bp A/T-rich sequence immediately preceding a site with a tandem repeat of two (A/G)GGTCA motifs spaced by two nucleotides [4].

Our recent work has demonstrated that REV-ERB*α* suppresses chemokine (*C-C motif*) ligand 2 (*ccl2*) gene expression directly through a RORE in the* ccl2* promoter region [5]. These results implicate REV-ERB*α* as a critical intermediary between the core clockwork and inflammatory pathways. Gibbs et al. [6] have demonstrated that the administration of a REV-ERB*α* ligand or a genetic knockdown of* rev-erb* *α* expression is effective at modulating the production and release of the proinflammatory cytokine interleukin-6 (IL6). Furthermore, Journiac and coworkers [7] have shown that 2 and 3 putative ROREs have also been found in the* il6* promoter region of mice and rats, respectively [7]. However, it is unclear whether the putative ROREs in the murine* il6* promoter are sensitive to REV-ERB*α* regulation. In some cases, there are several similarities and differences in the inflammatory response to endotoxin in mice and humans [8]. Therefore, it is important to demonstrate the impact of REV-ERB*α* on* il6* gene in murine immune cells as well as humans.

Results from the current study showed that REV-ERB*α* directly and indirectly suppresses* il6* gene expression in macrophages through a RORE and a nuclear factor *κ*B response element (NF*κ*BRE), respectively, in the murine* il6* promoter region. Furthermore, we observed increases in* il6* gene expression in peritoneal macrophages from mice lacking* rev-erb* *α*. REV-ERB*α* may therefore be a key link between the clockwork and inflammation.

## 2. Materials and Methods

### 2.1. Animals

C57BL/6J mice and B6.Cg-Nr1d1<tm1Ven>/LazJ mice were obtained from Sankyo Labo Service (Tokyo, Japan) and Jackson Laboratories (Bar Harbor, ME), respectively. The mice were housed in plastic cages and reared at 23°C with a 12 h light/dark cycle. Food and water were available ad libitum. All animals were cared for in accordance with the Guiding Principles for the Care and Use of Animals approved by the Council of the Physiological Society of Japan, based upon the Declarations of Helsinki, 1964.

### 2.2. Preparation and Culture of Peritoneal Macrophages

Peritoneal macrophages were collected from 2-month-old C57BL/6J mice and* rev-erb* *α* ^*−/−*^ mice and cultured as described previously [5, 9–11]. The cells from C57BL/6J mice were treated with or without REV-ERB*α* agonist, 2 or 20 *μ*M GSK4112 (Sigma Aldrich), for 16 h in the absence or presence of 1 *μ*g/mL lipopolysaccharide (LPS) from* Escherichia coli* 055 (Sigma Aldrich, St. Louis, MO). GSK4112 was dissolved with DMSO, and the control cells were treated using the same volume of DMSO. The cells from* rev-erb* *α* ^*−/−*^ mice were stimulated with or without LPS for 24 h.

### 2.3. Cell Line Culture

The murine macrophage cell line RAW264 (RCB0535) was purchased from RIKEN Cell Bank (Ibaraki, Japan) and cultured as described previously [5, 9, 10, 12]. To study the effects of REV-ERB*α* agonists on* il6* gene expression, the cells were treated with or without 20 *μ*M GSK4112 or 1 *μ*g/mL LPS for 16 h.

### 2.4. Real-Time Quantitative PCR (qPCR)

Total cellular RNA was prepared from peritoneal macrophages using an RNeasy Mini Kit (Qiagen, Hilden, Germany) and from RAW264 cells using RNAiso reagent (Takara bio, Siga, Japan). Extracted RNA was reverse-transcribed using a High-Capacity cDNA Reverse Transcription Kit (Applied Biosystems, Foster City, CA) with random primers. The reaction mixture was amplified in a Power SYBR Green Master Mix (Applied Biosystems) using a 7500 Real-Time PCR System (Applied Biosystems) with 200 nM oligonucleotide primers (forward and reverse). The oligonucleotide sequences used for qPCR were as follows:* il6*: 5′-GAT GGA TGC TAC CAA ACT GGA-3′ (forward), 5′-CCA GGT AGC TAT GGT ACT CCA GAA-3′ (reverse); *β* *actin* (*actb*, internal control), 5′-AAG GCC AAC CGT GAA AAG AT-3′ (forward), and 5′-GTG GTA CGA CCA GAG GCA TAC-3′ (reverse). The expression of the target gene was normalized to the housekeeping gene* actb*.

### 2.5. rev-erb*α* or ror*α* Plasmid Constructs and Stable Transfection

A stable* rev-erb* *α* transfectant (RAWrev) and the control cell line (RAWvecB) and a stable* ror* *α* transfectant (RAWror) and the control cell line (RAWvecA) were established as described previously [5].

### 2.6. Western Blot Analysis

Nucleic and cytosolic protein was extracted as described previously [5, 10, 13]. Extracted proteins were separated by SDS-PAGE and then transferred to a polyvinylidene difluoride membrane (Millipore, Milford, MA). Membranes were blocked with 5% nonfat dried milk in TBST and then immunoblotted with rabbit polyclonal Abs against NF*κ*B p65 (sc-372, Santa Cruz Biothechnology, Santa Cruz, CA),*α*-Tublin (*α*-Tub, ab7291, Abcam, Cambridge, UK), or TATA binding protein (TBP, ab51841, Abcam). Thereafter, HRP-conjugated donkey anti-rabbit or anti-mouse IgG secondary Abs (GE Healthcare Japan, Tokyo, Japan) was applied. The immunoreactivity was visualized with an ECL reagent (Bio-Rad, Hercules, CA).

### 2.7. EMSA

Nuclear extracts were prepared as described [5, 10, 13]. The murine NF*κ*B consensus oligonucleotide probe (5′-AGT TGA GGG GAC TTT CCC AGG C-3′) was labeled with biotin. The nuclear protein (2.5 *μ*g) and labeled oligonucleotide probe (20 fmol) were incubated in 10 mM HEPES-KOH, pH 7.8, 50 mM KCl, 0.2 mM EDTA, 10% glycerol, 1 *μ*g poly(dI-dc), 0.05% NP-40, and 5 mM DTT at room temperature for 20 min, electrophoresed in 4.5% polyacrylamide gels, transferred onto a nylon membrane (Biodyne, Pall Corporation, Pensacola, FL), and UV cross-linked. To detect the signals, a Chemiluminescent Nucleic Acid Detection Module (Thermo Fisher Scientific, Rockford, IL) was used according to the manufacturer's protocol.

### 2.8. Luciferase Reporter Assay

For the analysis of the promoter activity of the NF*κ*B-responsive promoter reporter luciferase construct, the cells were transfected with pNF*κ*B-Luc (Clontech, Palo Alto, CA) using a LipofectAMINE Reagent (Invitrogen, Carlsbad, CA), and luciferase activity was determined using a Luciferase Assay System Kit (Promega, Madison, WI).

For the analysis of* il6* promoter activity, the murine* il6* promoter (distal fragment, −1029 to +31; proximal fragment, −649 to +31) was amplified from mouse genomic DNA (Promega) using an LA Taq polymerase (Takara bio) and was subcloned into pCR-XL-TOPO vector (Invitrogen). The subcloned fragments were digested at* Kpn*I/*Xho*I sites and cloned into pGL3 vector (Promega) at the corresponding sites. The cells were transiently transfected by using a LipofectAMINE Reagent with distal or proximal constructs containing the luciferase reporter gene, and luciferase activity was determined with a Dual Luciferase Assay System Kit (Promega). Activity was normalized relative to an internal cotransfected constitutive control (*Renilla* luciferase expression vector, pRL-TK vector, Promega), as described [5, 10, 12].

### 2.9. Mutagenesis

The* il6* promoter mutant construct was made by using a QuickChange Lightning Site-Directed Mutagenesis Kit (Stratagene, La Jolla, CA) as described [5, 12]. The proximal RORE (−529 to −518) was mutated from AAA CTC AGG  TCA to AAA CTC AGG  CCT by using the mutant primers 5′-CTG AAA AAA CTC AGG CCT GAA CAT CTG TAG-3′ (forward) and 5′-CTA CAG ATG TTC  AGG CCT GAG TTT TTT CAG-3′ (reverse) for the distal and proximal* il6* promoter constructs mutagenesis (underline, mutant sequences). The NF*κ*BRE (−91 to −82) was mutated from GGG  ATT  TTC C to GGG  CCC TTC C by using the mutant primers 5′-GAT TTT TAT CAA ATG TGG  GCC CTT CCC ATG AGT CTC-3′ (forward) and 5′-GAG ACT CAT GGG AAG GGC  CCA CAT TTG ATA AAA  ATC-3′ (reverse) for the proximal* il6* promoter constructs mutagenesis.

### 2.10. Statistical Analysis

The results were expressed as the means ± S.E. When two means were compared, a Student's *t*-test for unpaired samples was conducted. For more than two groups, the statistical significance of the data was assessed by ANOVA. When significant differences were found, individual comparisons were made between groups by using the *t*-statistic and adjusting the critical value according to the Tukey-Kramer method. Differences were considered significant at *P* < 0.05.

## 3. Results

### 3.1. REV-ERB*α* Agonists Suppress* il6* Induction following LPS Stimulation

**Figure 1:**
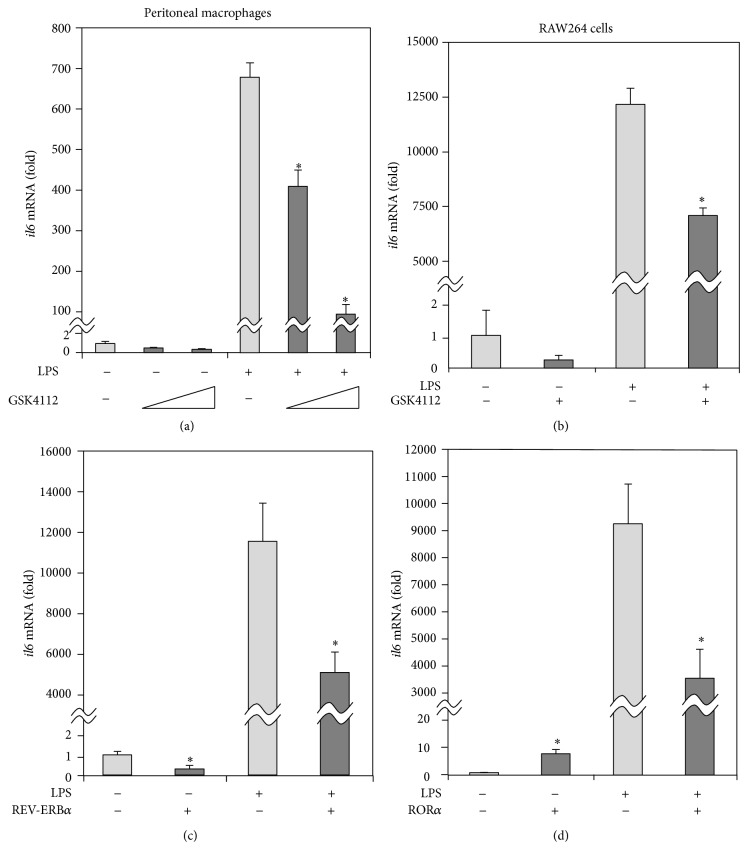
REV-ERB*α* represses* il6* gene induction following a LPS challenge in macrophages. (a) Peritoneal macrophages were harvested as adherent cells from 2-month-old C57BL/6J mice and were either untreated or treated with 1 *μ*g/mL LPS or 2 or 20 *μ*M GSK4112 for 16 h. (b) Murine macrophage cell line RAW264 cells were either untreated or treated with 1 *μ*g/mL LPS or 20 *μ*M GSK4112 for 16 h. (c) RAW264 cells transfected with or without* rev-erb* *α* were either untreated or treated with 1 *μ*g/mL LPS for 24 h. (d) RAW264 cells transfected with or without* ror* *α* were either untreated or treated with 1 *μ*g/mL LPS for 24 h. The gene expression of* il6* was analyzed by qPCR. For normalization,* actb* mRNA was used. The data are presented as the means ± S.E. (*n* = 3-4). ^*^ *P* < 0.05* versus* cells treated with LPS and without GSK4112 or* versus* vector control.

To determine the role of REV-ERB*α* in inflammatory responses, we analyzed the effects of the REV-ERB*α* agonist, GSK4112, on the gene expression of* il6* as a crucial inflammatory molecular element in macrophages. The induction of* il6* mRNA after LPS stimulation was dose-dependently repressed by the addition of GSK4112 in peritoneal macrophages (Figure 1(a)). Furthermore, as shown in Figure 1(b), qPCR analysis confirmed that GSK4112 treatment also decreased the induction of* il6* mRNA after LPS stimulation in murine macrophage cell line RAW264 cells as well as peritoneal macrophages. These data suggest that activation of REV-ERB*α* led to the suppression of* il6* gene induction in macrophages.

### 3.2. rev-erb*α* Overexpression Represses* il6* Expression

To investigate the potential role of REV-ERB*α* in* il6* expression in macrophages, a stable* rev-erb* *α* transfectant (RAWrev) and the control cell line (RAWvecB) were established using RAW264 cells [5]. As seen in Figure 1(c), overexpression of* rev-erb* *α* repressed the gene expression of* il6* in both the absence and presence of LPS, suggesting that REV-ERB*α* was involved in the suppression of* il6* gene expression in macrophages.

### 3.3. ror*α* Overexpression Enhances* il6* Expression

REV-ERB*α* is known to cross-talk with ROR*α* (orphan nuclear receptor encoded by* nr1f1*), another of the clock genes that has similar DNA binding specificity to REV-ERB*α* and competes for the binding of REV-ERB*α* [14–16]. Whereas REV-ERB*α* represses transcription from these sites of the target genes, ROR*α* acts as a transcriptional activator [3, 4, 17]. From these findings, we hypothesized that ROR*α* might positively regulate* il6* expression and established a stable* ror* *α* transfectant (RAWror) and the control cell line (RAWvecA) using RAW264 cells [5]. Interestingly, overexpression of* ror* *α* enhanced the gene expression of* il6* in the absence of LPS, whereas it repressed the gene expression of* il6* in the presence of LPS (Figure 1(d)), indicating that regulation of* il6* gene expression by ROR*α* is different between nonactivated and activated states in macrophages.

### 3.4. REV-ERB*α* Suppressed NF*κ*B Activity

**Figure 2:**
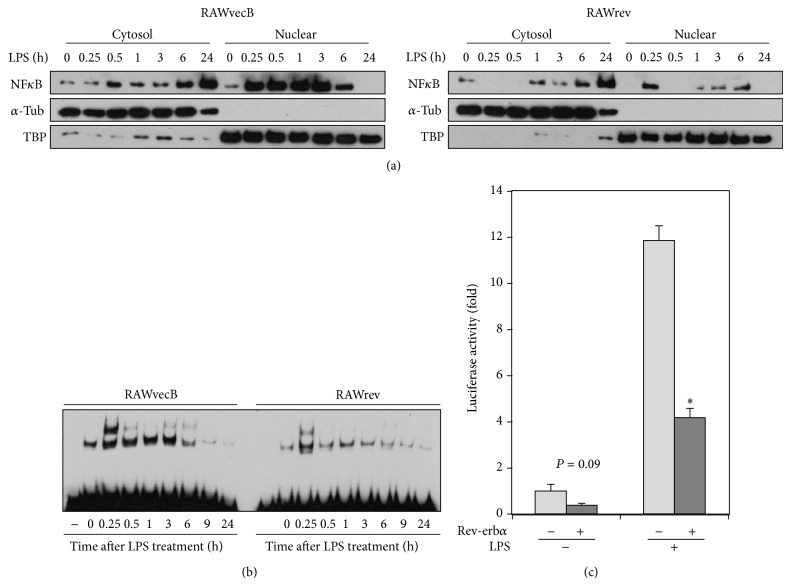
REV-ERB*α* represses NF*κ*B activation in macrophages. (a) Nuclear and cytoplasmic fractions of each cell untreated or treated with LPS for various durations were analyzed by Western blot for p65 NF*κ*B,*α*-Tub, and TBP. Detection of*α*-Tub and TBP is used as marker of nuclear and cytoplasmic fractions, respectively. (b) Each cell was stimulated with LPS for varying durations and NF*κ*B activation was analyzed by EMSA. Data shown are representative of three separate experiments. (c) Each cell was transiently transfected with NF*κ*B-responsive promoter reporter luciferase construct and luciferase activities in each cell stimulated either with or without LPS for 24 h were analyzed. The data are presented as the means ± S.E. from sextuplicate cultures. ^*^ *P* < 0.01* versus* vector control.

The* il6* gene contains a functional *κ*B element in its promoter region [18]. Thus, activation of NF*κ*B leads to the transcription of this proinflammatory gene. Therefore, we next investigated whether REV-ERB*α* regulates NF*κ*B activity in macrophages. As shown in Figure 2(a), cytosolic expression and LPS-induced nuclear translocation of NF*κ*B subunit p65 were attenuated in RAWrev cells, compared with RAWvecB cells. No marked change in inhibitory *κ*B (I*κ*B) expression was observed between RAWrev and RAWvecB cells (data not shown). Furthermore, LPS-induced NF*κ*B activation in RAWrev cells was markedly lower than that in RAWvecB cells (Figure 2(b)). In addition, REV-ERB*α* attenuated the promoter activity of the NF*κ*B-responsive promoter reporter luciferase construct in both the absence and presence of LPS in macrophages (Figure 2(c)). These results strongly suggest that REV-ERB*α* suppresses LPS-enhanced NF*κ*B activity in macrophages.

### 3.5. REV-ERB*α* Represses the Activity of the Murine* il6* Promoter

**Figure 3:**
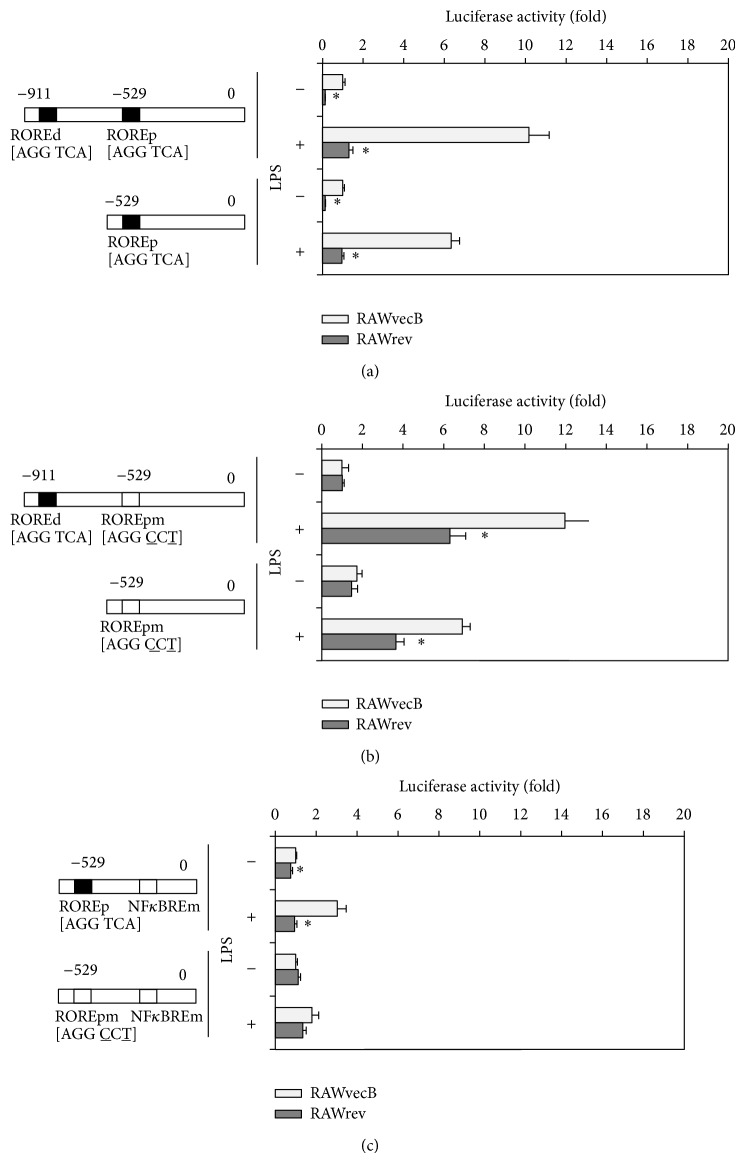
REV-ERB*α* represses* il6* promoter activity, independent of the inhibition of NF*κ*B signaling. (a) RAWrev and RAWvecB cells were transiently transfected with luciferase reporter construct containing either a distal or a proximal construct of* il6* promoter. After treatment with or without 1 *μ*g/mL LPS for 24 h, luciferase activities were determined. ROREd, distal RORE; ROREp, proximal RORE. (b) The AGGTCA half-site in the proximal RORE was changed to AGGCCT by site-directed mutagenesis of nucleotides −518 (A to T) and −520 (T to C), and luciferase activities of each cell either untreated or treated with LPS for 24 h were determined. ROREpm, proximal RORE mutant. (c) The GGGATTTTCC half-site in the NF*κ*BRE was changed to GGGCCCTTCC by site-directed mutagenesis of nucleotides −86 (T to C), −87 (T to C), and −88 (A to C), and luciferase activities of each cell either untreated or treated with LPS for 24 h were determined. NF*κ*BREm, NF*κ*BRE mutant. Luciferase values were normalized using* Renilla* luciferase. The data are presented as the means ± S.E. from sextuplicate cultures. ^*^ *P* < 0.05* versus* vector control.

Two putative ROREs have been found in the mouse* il6* promoter sequence [7]. Therefore, to determine whether the putative ROREs in the* il6* promoter are sensitive to REV-ERB*α* regulation, we cloned* il6* promoters with different lengths—a distal promoter that included one RORE located in the distal region and one RORE located in the proximal region, and a proximal promoter that included one RORE located in the proximal region—into a luciferase reporter vector. Then, these two constructs were transiently transfected into cell lines, RAWrev and RAWvecB cell lines. The activities of each longitudinal promoter in RAWrev cells were considerably lower than those in RAWvecB cells in both the absence and presence of LPS (Figure 3(a)). We next investigated whether two ROREs in the* il6* promoter are necessary for REV-ERB*α*-mediated repression. As shown in Figure 3(b), the mutation of the proximal RORE abolished the repression of the promoter activities in RAWrev transfected with the distal construct as well as the proximal construct in the absence of LPS. These results suggest a critical role for the proximal RORE in REV-ERB*α*-mediated repression of* il6* expression. However, the mutation of the proximal RORE still repressed the promoter activities in RAWrev transfected with the distal construct as well as the proximal construct in the presence of LPS (Figure 3(b)), indicating that REV-ERB*α* repressed* il6* promoter activity through other transcriptional regulators such as NF*κ*B, which was independent of the direct binding of REV-ERB*α* to the RORE in the promoter.

### 3.6. REV-ERB*α* Represses* il6* Promoter Activity, Independent of NF*κ*B

To dissect the effect of NF*κ*B on* il6* promoter activity, we used point-mutated variants in the response element of NF*κ*B. The activity of an* il6* promoter containing an NF*κ*BRE mutated construct in RAWrev cells was lower than that in RAWvecB cells in both the absence and presence of LPS (Figure 3(c)). These results show that REV-ERB*α* repressed* il6* promoter activity, independent of NF*κ*B. A double mutation of RORE and NF*κ*BRE completely abrogated the suppression of the promoter activity in RAWrev cells in both the absence and presence of LPS (Figure 3(c)). These results suggest both a direct and an indirect repression of the* il6* promoter activity by REV-ERB*α*.

### 3.7. ROR*α* Enhances the Activity of Murine* il6* Promoter

**Figure 4:**
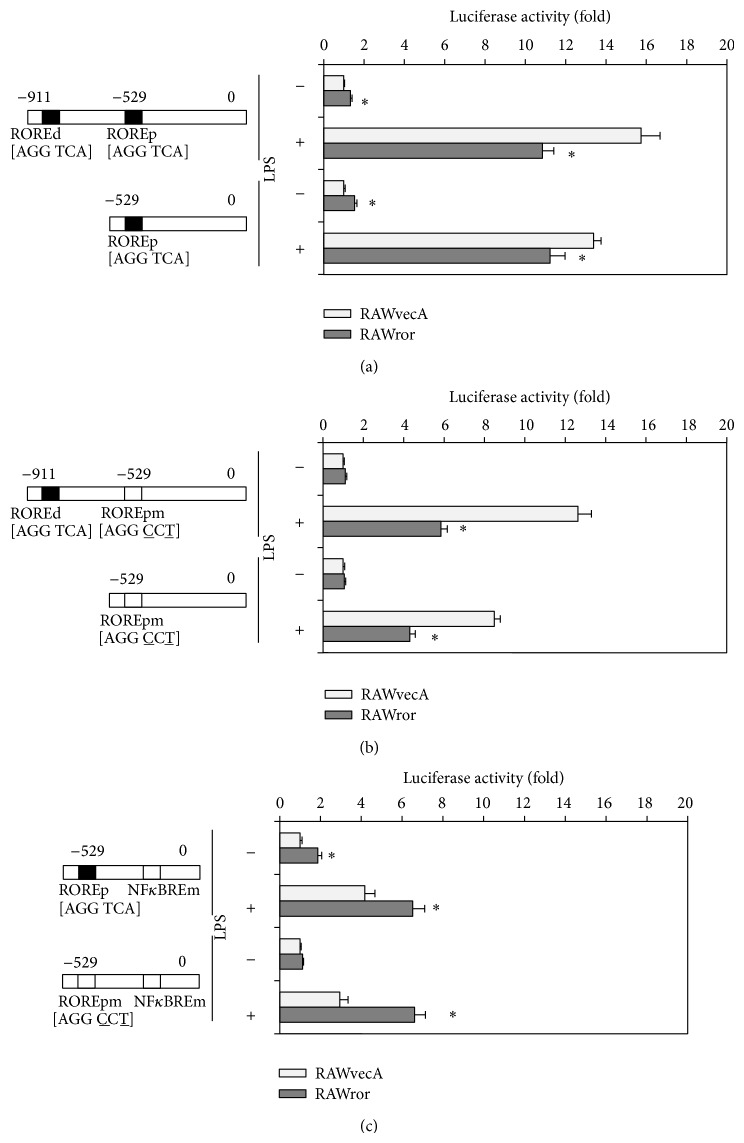
Effect of ROR*α* on* il6* promoter activity. (a) RAWror and RAWvecA cells were transiently transfected with luciferase reporter construct containing either a distal or a proximal construct of* il6* promoter. After treatment with or without 1 *μ*g/mL LPS for 24 h, luciferase activities were determined. ROREd, distal RORE; ROREp, proximal RORE. (b) The AGGTCA half-site in the proximal RORE was changed to AGGCCT by site-directed mutagenesis of nucleotides −518 (A to T) and −520 (T to C), and luciferase activities of each cell either untreated or treated with LPS for 24 h were determined. ROREpm, proximal RORE mutant. (c) The GGGATTTTCC half-site in the NF*κ*BRE was changed to GGGCCCTTCC by site-directed mutagenesis of nucleotides −86 (T to C), −87 (T to C), and −88 (A to C), and luciferase activities of each cell either untreated or treated with LPS for 24 h were determined. NF*κ*BREm, NF*κ*BRE mutant. Luciferase values were normalized using* Renilla* luciferase. The data are presented as the means ± S.E. from sextuplicate cultures. ^*^ *P* < 0.05* versus* vector control.

Because ROR*α* activates target genes via ROREs in their promoters, we reasoned that ROR*α* might positively regulate* il6* promoter activity. Therefore, we transiently transfected the distal and the proximal* il6* promoter constructs into RAWror and RAWvecA cells. The activity of each of the liner promoter in the RAWror cells was considerably higher than that in RAWvecA cells in the absence of LPS, whereas that in RAWror cells was lower than that in RAWvecA cells in the presence of LPS (Figure 4(a)). We also investigated whether two ROREs in the* il6* promoter were essential for ROR*α*-mediated enhancement of* il6* expression. The mutation of a proximal RORE abrogated the enhancement of the promoter activities in RAWror cells transfected with either distal or proximal construct in the absence of LPS (Figure 4(b)), suggesting that the positive regulatory effects of ROR*α* on the* il6* expression are mainly dependent on the proximal RORE in the* il6* promoter. However, the mutation of the proximal RORE additionally repressed the promoter activities in RAWror transfected with the distal construct as well as the proximal construct in the presence of LPS (Figure 4(b)). Therefore, we hypothesized that ROR*α* also suppressed* il6* promoter activity via the inhibition of NF*κ*B-induced transactivation after LPS stimulation as is the case with REV-ERB*α*. In fact, the activity of an* il6* promoter containing an NF*κ*BRE mutated construct in RAWror cells was higher than that in RAWvecA cells in both the absence and presence of LPS (Figure 4(c)). These results show that ROR*α* activated* il6* promoter activity through proximal RORE in nonactivated cells, whereas it indirectly repressed the activity through negative regulation of NF*κ*B signaling in activated cells. A double mutation of RORE and NF*κ*BRE showed no changes in the promoter activity between RAWror and RAWvecA cells in the absence of LPS (Figure 4(c)). However, in the presence of LPS,* il6* promoter activity of RAWror cells is lower than that of RAWvecA cells, suggesting that ROR*α* also repressed* il6* promoter activity through other transcriptional regulators than NF*κ*B.

### 3.8. Peritoneal Macrophages from rev-erb*α* Knockout Mice Display Increases in the* il6* Gene Expression

**Figure 5:**
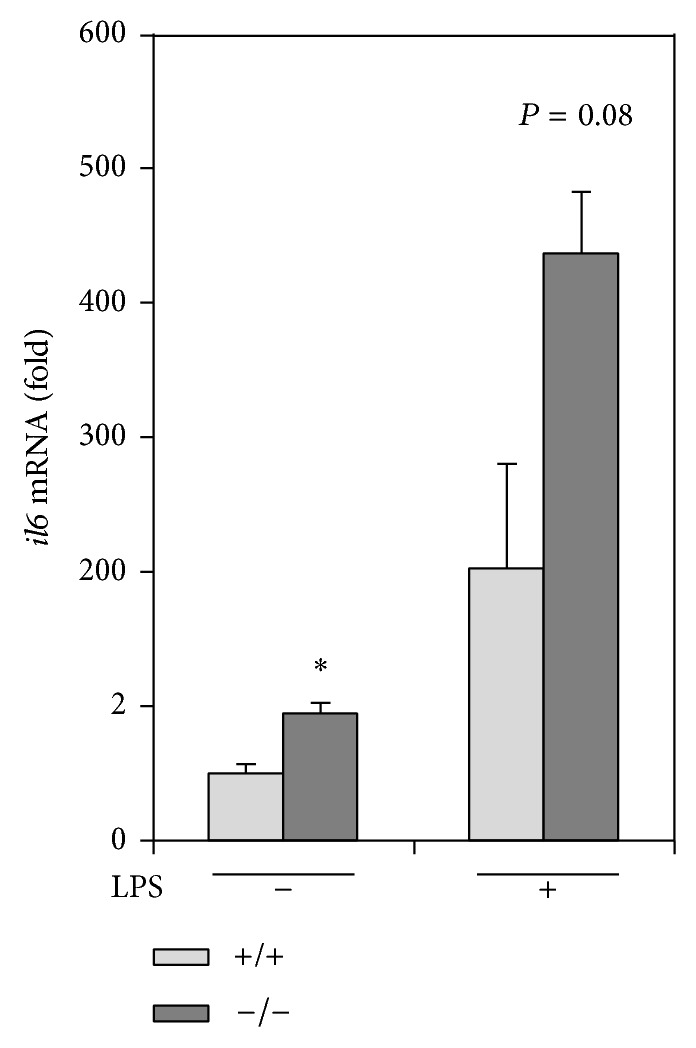
Peritoneal macrophages from* rev-erb* *α* ^*−/−*^ mice increase* il6* gene expression. Peritoneal macrophages were harvested as adherent cells from 2-month-old* rev-erb* *α* ^*−/−*^ mice and their wild-type (+/+) mice. The cells were treated with or without 1 *μ*g/mL LPS for 24 h. The gene expression of* il6* was analyzed by qPCR. For normalization,* actb* mRNA was used. The data are presented as the means ± S.E. (*n* = 3). ^*^ *P* < 0.05* versus rev-erb* *α* ^*+/+*^ mice.

To test whether results observed in the* in vitro* study are physiologically relevant, we investigated the effects of a* rev-erb* *α* deficiency on* il6* expression in peritoneal macrophages using* rev-erb* *α* ^*−/−*^ mice. As shown in Figure 5,* il6* gene expression in the absence of LPS in the peritoneal macrophages of* rev-erb* *α* ^*−/−*^ mice was significantly higher than that in wild-type mice. The induction of the* il6* gene following a LPS challenge in the peritoneal macrophage of mice lacking* rev-erb* *α* also was relatively higher (*P* = 0.08) than that found in wild-type mice. These results show that* il6* expression is negatively regulated by REV-ERB*α* *in vivo* as well as* in vitro*.

## 4. Discussion

Until recently, REV-ERB*α* was considered to be a constitutively active nuclear orphan receptor, although heme has now been shown to bind reversibly to the receptor and to drive ligand-dependent activity [19]. This implies that REV-ERB*α* is responsive to the cellular redox state and perhaps to gaseous signaling molecules such as NO and CO through interactions with heme [20]. In addition, REV-ERB*α* acts as a transrepressor for a number of genes, including* bmal1* [14],* apolipoprotein AI* (*apoAI*) [21],* apoCIII* [22],* fibrinogen-β* [23],* plasminogen activator inhibitor type 1* (*pai1*) [24],* il6* [7], and* ccl2* [5], which indicates that the nuclear hormone receptor plays an important role in the regulation of metabolism, the cardiovascular system, and inflammation.

Recently, we demonstrated that REV-ERB*α* negatively regulates the inflammatory function of macrophages through the direct repression of* ccl2* expression [5]. Furthermore, we showed that REV-ERB*α* suppresses not only intracellular signals such as extracellular signal-regulated protein kinase (ERK) and p38 mitogen-activated protein kinase (p38 MAPK), which is known as CCL2 and the receptor chemokine (C-C motif) receptor 2- (CCR2-) activated signaling pathways, but also the inflammatory functions of macrophages such as adherent and migratory activities, the activation of which is known to be dependent on CCL2-mediated ERK and p38, respectively [5, 25]. These observations identified the nuclear hormone receptor REV-ERB*α* as an anti-inflammatory receptor and a therapeutic target in inflammatory disease.

As in the previous report, for the current study, we analyzed the role of REV-ERB*α* in the gene expression of inflammatory cytokine* il6* in murine macrophages. We confirmed that REV-ERB*α* agonist GSK4112 inhibits the induction of the* il6* gene in murine peritoneal macrophages and in murine macrophage cell line RAW264 cells following LPS stimulation. Our results are consistent with the recently published results by Gibbs et al. [6] who demonstrated that GSK4112 abolishes the induction of inflammation-related genes, including* il6*, following a LPS challenge, using primary human monocyte-derived macrophages. In the current study, the overexpression of* rev-erb* *α* also revealed that REV-ERB*α* contributes to the negative regulation of* il6* expression in macrophages. By contrast, peritoneal macrophages from mice lacking* rev-erb* *α* increase* il6* gene expression. Reporter assay and site-directed mutagenesis identified a critical role for the proximal RORE in the murine* il6* promoter in REV-ERB*α*-mediated repression of* il6* expression. We also showed that REV-ERB*α* represses* il6* expression not only directly through a RORE but also indirectly through an NF*κ*BRE in the murine* il6* promoter. These results strongly suggest that REV-ERB*α* functions as a repressor of inflammatory response in macrophages via the inhibition of the target genes, including* ccl2* and* il6*.

REV-ERB*α* has been known to cross-talk with ROR*α*, an orphan nuclear receptor encoded by* nr1f1*, that has similar DNA binding specificity to REV-ERB*α*, acts as a constitutive transcriptional activator, and thus competes with the binding of REV-ERB*α* [3, 4, 14–17, 26]. Furthermore, Journiac and coworkers [7] have shown that REV-ERB*α* and/or ROR*α* directly bind to a RORE in the human* il6* promoter and acts as a transrepressor and a transactivator of* il6* gene expression, respectively. We also confirmed that ROR*α* overexpression in murine macrophage cell line enhances* il6* gene expression and the promoter activity through RORE in its promoter region without any exogenous LPS stimulation, whereas it suppressed* il6* gene induction and the promoter activity, at least partly, via the inhibition of NF*κ*B-induced transactivation after LPS stimulation. These results suggest that ROR*α* transactivates* il6* expression by interacting with a RORE in the promoter of murine macrophages, whereas ROR*α* negatively regulates* il6* expression through the NF*κ*B signaling in murine macrophages. From these observations, in resting cultured macrophages, a dual regulation also pertains to the* il6* promoter activity; REV-ERB*α* potently represses* il6* promoter activity, whereas ROR*α* potently enhances* il6* promoter activity through murine* il6* promoter as well as human [7]. The dual regulation seems to have an advantage in the modulation of inflammatory responses of macrophages, although evidence for* in vivo* relevance is clearly lacking.

Patients with rheumatoid arthritis (RA) report daily variations in their symptoms, experiencing greater joint pain, stiffness, and functional disability in the mornings, which is accompanied by fluctuations in circulating IL6 concentration [27, 28]. Some asthma patients experience nighttime exacerbations that can be attributed to not only daily variations in lung physiology but also increased bronchial responsiveness at night [29]. Macrophages exhibit a rhythmic expression of* rev-erb* *α* , are capable of cell-autonomous gene oscillation in culture, and display a robust circadian gating in their responses to exogenous inflammatory stimulation [6, 30–32]. Therefore, it seems likely that REV-REB*α* expressed in macrophages plays an important role in the regulation of the diurnal fluctuation of several inflammatory diseases as well as in the production and secretion of these inflammatory and/or anti-inflammatory factors. Further studies are needed to clarify the* in vivo* relationship between REV-ERB*α* and inflammatory diseases. Taken together, the results of the current study indicate that REV-ERB*α* is a potent anti-inflammatory receptor and a therapeutic target for inflammatory diseases.

## 5. Conclusion

We demonstrated that a circadian clock gene, REV-ERB*α*, represses* il6* expression not only indirectly through an NF*κ*B binding motif but also directly through a REV-ERB*α* binding motif in the murine* il6* promoter region. Overexpression of* rev-erb* *α* in murine macrophage cell line suppressed* il6* induction and NF*κ*B activity following a LPS endotoxin challenge. The present study also showed that peritoneal macrophages from mice lacking* rev-erb* *α* display increases in* il6* expression. These data suggest that REV-ERB*α* regulates the inflammatory response of macrophages through the suppression of* il6* expression. REV-ERB*α* may therefore be a key link between clockwork and inflammation.

## Abbreviations

NF*κ*BRE: Nuclear factor *κ*B response element
ROR: Retinoic acid receptor-related orphan receptor
RORE: ROR response element.
